# Semi-Physical Simulation Method for Stellar Maps with Color Temperature Information

**DOI:** 10.3390/s25123737

**Published:** 2025-06-14

**Authors:** Yu Zhang, Bin Zhao, Ke Zhang, Jian Zhang, Songzhou Yang, Dongpeng Yang, Taiyang Ren, Dianwu Ren, Junjie Yang, Jingrui Sun

**Affiliations:** 1 State Key Laboratory of High Power Semiconductor Lasers, Changchun University of Science and Technology, Changchun 130022, China; 2 School of Opto-Electronic Engineering, Changchun University of Science and Technology, Changchun 130022, China

**Keywords:** stellar map, color temperature simulation, semi-physical simulation, star sensor, color temperature deviation calibration

## Abstract

Existing stellar map simulators lack color temperature information, have complex system structures, and cannot independently control the color temperatures of stars. Therefore, this study developed an OLED-based semi-physical simulation method and a simulation algorithm for stellar maps with color temperature information to realize a semi-physical simulation of stellar maps close to the real situation in space. The study also aimed to independently control the color temperature of each star. The simulation effect of the stellar map with color temperature information was verified using four stellar maps. The developed simulator achieved independent and controllable color temperature information for each star in the stellar map.

## 1. Introduction

The star sensor—a star-sensitive device—is a key component of the spacecraft attitude determination and control system whose accuracy directly affects the performance of the navigation system [1]. The working conditions of star-sensitive devices [2] have become increasingly complex, and navigation methods have developed from traditional astronomical angular measurements or ranging navigation methods to emerging navigation technologies such as astronomical spectral velocimetry navigation using stellar color temperature information [3,4,5]. Existing star simulators, which can only provide limited information on the color temperatures of stars [6,7], fail to satisfy the ground-based calibration requirements of star-sensitive devices.

Recent efforts have been made to accurately simulate the multi-dimensional information of stellar maps in outer space. For example, Linghao Wu et al. [8] developed a star simulator with stray light suppression capability and simulated stellar maps of stars as dim as magnitude +6 Mv. Schulz et al. [9] developed a stellar map generation method with controllable Gaussian background noise and simulated stellar map with an approximate sky background. Teague and Chahl [10] developed a simulation method for motion-blurred stellar maps, effectively correcting the grayscale information of stellar maps in stellar map display devices through color space conversion methods. Although this method only generated grayscale stellar maps, it has significant implications for simulating stellar maps with color temperature information using a single stellar map display device.

With the development of spectral simulation architectures from multi-LED mixing [11] to spatial light modulation-based dimming [12], stellar spectral simulation methods have been extensively investigated. Liu et al. [13] simulated a single star in the 3000–11,000 K color temperature range using a digital micromirror device (DMD) and a fuzzy proportional–integral–derivative (PID) control algorithm. Yun et al. [14] extended the color temperature simulation range to 2000–12,000 K by combining backpropagation (BP), a neural network, and a PID control algorithm. Although the above two methods can simulate the color temperature information of individual stars more realistically, they cannot simulate the real state of stars with different color temperature information in a stellar map.

To improve the similarity between stellar maps and the actual state of outer space, Bin Zhao et al. [15] proposed a fast playback method in which the stellar maps were fused with cosmic background radiation information to improve the stellar maps’ refreshing speed. However, the simulation of stellar maps with stellar color temperature information has still not been realized. In 2024, Professor Niu Zhaodong’s team at the National University of Defense Technology in China proposed a two-step matching algorithm for stellar map matching problems such as high-limit stars. Under the problem of unreliable luminance information caused by background interference, such as clouds and Earth’s atmospheric radiation, the matching strategy relied solely on angular features to avoid the interference of luminance information [16]. In 2021, Wang Hongyuan and others proposed a simulation model based solely on magnitude by introducing the stellar color index and blackbody radiation theory in response to background interferences such as cosmic radiation, nebula and star cluster noise, and stray light. This improvement significantly enhances the robustness and accuracy of star map simulation when dealing with background interference [17]. Songzhou Yang et al. [18] developed a “series–parallel” hybrid, multi-source, information fusion, spatial target simulation system. In this system, three light sources (one LED light source and two blackbody light sources) and three spatial light modulation devices (one thin-film transistor liquid crystal display (TFT-LCD) and two DMDs) were used to simulate each star in the stellar map. The energy ratio of four spectral intervals (UV, visible, mid-wave infrared, and long-wave infrared) of each star in the stellar map could be adjusted. However, the system structure was complicated, and the system could not accurately simulate the color temperature.

In summary, existing stellar map simulators have various shortcomings, such as missing color temperature information, complex system structure, and inability to independently control the color temperature of stars. Therefore, this study aimed to address this research gap by developing a semi-physical stellar map simulation method with color temperature information. The stellar map simulator leveraged an OLED-based architecture. A stellar map simulation link with color temperature information was established, and a stellar map generation algorithm with color temperature information was proposed. We successfully controlled the color temperature of each star in the semi-physical simulation in the stellar map. This study provides new technical means and implementation approaches for the ground calibration and testing of star sensors. The developed star simulator has important theoretical and practical significance.

## 2. Framework for Semi-Physical Simulations of Stellar Maps with Color Temperature Information

When significantly distant stars are observed through optical payloads such as star sensitizers, they can be regarded as point light sources at infinity [19]. Currently, star simulators use liquid crystal on silicon (LCOS) [20], LCD [21], or DMD [22] as stellar map display devices placed at the focal plane, in accordance with the working principle of the collimated optical system [23]. Combined with the stellar map illumination device [24], stellar maps are simulated at infinity by loading the grayscale images of stellar maps. However, the current stellar map illumination devices are mostly LED light sources [25], blackbody radiation sources [26], or specially designed illumination optical systems [27], which can only output illumination beams with fixed color temperature information. The illumination area of the stellar map illumination device covers the entire range of the stellar map display device. Therefore, the stars displayed in existing stellar map display devices all have the same color temperature information. However, the stars in actual space have different color temperature information, resulting in inconsistency between the simulated and actual color temperature information. Therefore, in this study, the architecture of the star simulator is simplified by replacing the traditional stellar map display device and stellar map illumination device with a self-luminous OLED device that enables independent and controllable color temperature information of each pixel. As such, the stellar map simulation with color temperature information is realized so that the stellar map simulated is closer to the actual state of stars in the universe.

**Figure 1:**
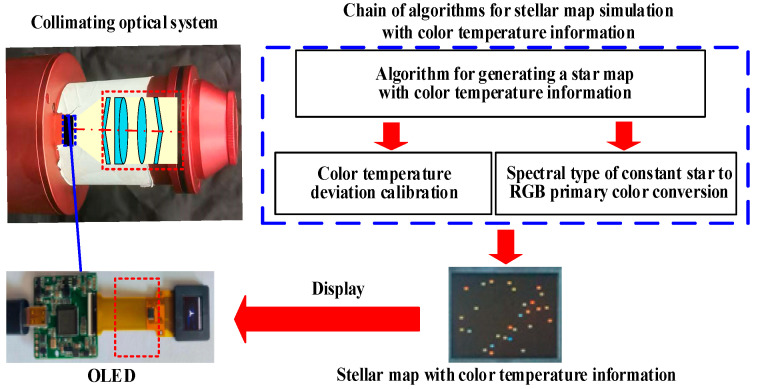
Architecture and algorithmic links for simulating stellar maps with color temperature information.

The color temperature information of stars is primarily classified based on their surface temperatures [28], and the OLED devices adjust the color temperature by adjusting the ratio of the luminous intensity of the three primary colors of red (R), green (G), and blue (B) [29]. The light beam emitted from the stellar map displayed by the OLED device passes through the collimated optical system. Thus, the color temperature of the simulated stellar map deviates from that of the OLED device [30] and the spectral transmittance of the collimated optical system [31]. Compensating for this combined color temperature deviation is necessary. Therefore, a stellar map simulation algorithm linked with color temperature information was formulated to improve the ability of the OLED-based stellar map simulation architectures to accurately simulate stellar maps containing color temperature information. The stellar map simulation architecture and algorithm link with color temperature information are shown in Figure 1.

## 3. Algorithm for Stellar Map Simulation with Color Temperature Information

**Figure 2:**
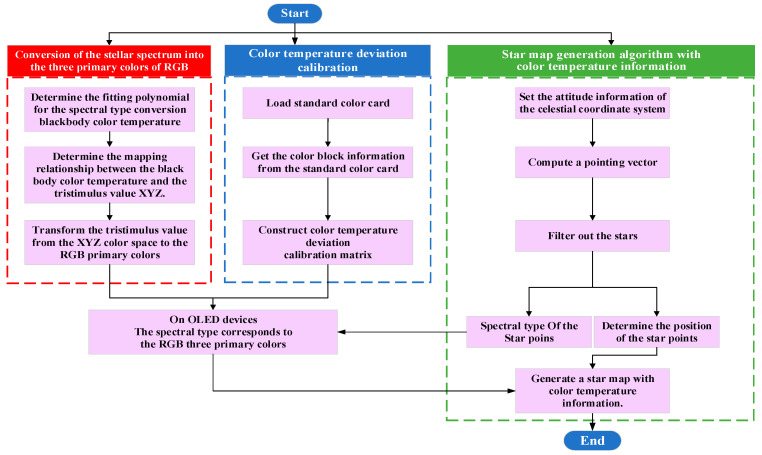
Algorithm flow of stellar nap simulation with color temperature information.

According to the architecture and algorithmic links, the stellar map simulation algorithm with color temperature information was divided into three modules: conversion of the stellar spectrum into the three primary RGB colors, color temperature deviation calibration, and the stellar map generation algorithm with color temperature information. The algorithmic flow is shown in Figure 2.

### 3.1. Stellar Spectral Type to RGB Triple-Color Conversion

Stellar spectral types are primarily classified into seven categories according to the Harvard Spectral Classification (HSC): O, B, A, F, G, K, and M. Each spectral type is further subdivided into ten subtypes, ranging from 0 to 9 [32]. No formula has been established to convert stellar spectral types directly to RGB triplet colors owing to the complexity of stellar spectra and the nonlinear relationship between spectral features and blackbody color temperature. Therefore, the stellar spectral types were numerically coded to facilitate calculation and analysis, with 1 as the coding interval. The 70 spectral subtypes from O0 to M9 were sequentially coded from 00 to 69 starting from 0. The coding rules are listed in Table 1.

Based on the discrete data of stellar spectral type and temperature provided by Harre and Heller [33], the spectral type of the star was converted to the corresponding stellar temperature using a polynomial fitting method [34], given as follows:

(1) $$f(s)=b_{1}\cdot s+b_{2}\cdot s^{2}+\ldots b_{n}\cdot s^{n}$$

where f(s) is the stellar temperature, s is the spectral-type code, and *b*_1_, *b*_2_, …, *b*_n_ are the polynomial coefficients corresponding to the nth spectral-type code. As stellar radiation approximates blackbody radiation at a certain temperature [35], according to Planck’s formula for blackbody radiation, the spectral profile emitted by a star can be expressed as follows:

(2) $$E(\lambda ,f(s))=\frac{2hc^{2}}{\lambda^{5}}\cdot \frac{1}{e^{\frac{hc}{\lambda kf(s)}}-1}$$

where *λ* is the wavelength, *h* is Planck’s constant ($h=6.626\times 10^{-34}J\cdot s$), *c* is the speed of light ($c=3\times 10^{8}m/s$), and *k* is Boltzmann’s constant ($k=1.381\times 10^{-23}J/K$). According to the color matching functions, $\bar{x}(\lambda )$, $\bar{y}(\lambda )$, and $\bar{z}(\lambda )$ are defined by the International Commission on Illumination (CIE) [36]. The triple-stimulus XYZ values of the stellar spectral curve [37] were obtained as follows:

(3) $$\left\{\begin{array}{c} X=\int_{380}^{780}E(\lambda ,f(s))\cdot \bar{x}(\lambda )d\lambda \\ Y=\int_{380}^{780}E(\lambda ,f(s))\cdot \bar{y}(\lambda )d\lambda \\ Z=\int_{380}^{780}E(\lambda ,f(s))\cdot \bar{z}(\lambda )d\lambda \end{array}\right.$$

The corresponding RGB triple-base color on the OLED was obtained through the RGB conversion matrix [38] as follows:

(4) $$\left[\begin{array}{c} R\\ G\\ B \end{array}\right]=\left[\begin{array}{ccc} a_{11} & a_{12} & a_{13}\\ a_{21} & a_{22} & a_{23}\\ a_{31} & a_{32} & a_{33} \end{array}\right]\cdot \left[\begin{array}{c} X\\ Y\\ Z \end{array}\right]$$

where $a_{ij}$ is an element of the RGB transformation matrix representing the linear transformation coefficients from the XYZ to RGB color spaces.

### 3.2. Color Temperature Deviation Calibration

The color temperature deviation calibration was performed by loading the standard color card images of multiple color blocks on the OLED device and using a color temperature-calibrated industrial camera to obtain the three chromatic colors of each color block after collimating the optical system. Thus, the color temperature deviation calibration matrix was obtained.

Let the number of color blocks in the standard color card be n. The theoretical tri-color of the standard color block numbered i can be expressed as ${\left[\begin{array}{ccc} R_{i} & G_{i} & B_{i} \end{array}\right]}^{T}$, where $i\in \left[1,n\right]$. Then, the theoretical RGB tri-color matrix of the standard color card is 

$RGBP=\left[\begin{array}{cccc} R_{1} & R_{2} & \ldots & R_{n}\\ G_{1} & G_{2} & \ldots & G_{n}\\ B_{1} & B_{2} & \ldots & B_{n} \end{array}\right]$. The industrial camera can obtain the RGB tri-color of the corresponding standard color block as ${\left[\begin{array}{ccc} {R^{\prime }}_{i} & {G^{\prime }}_{i} & {B^{\prime }}_{i} \end{array}\right]}^{T}$. Then, the RGB tri-color matrix of the standard color card obtained by the industrial camera is $RGBM=\left[\begin{array}{cccc} {R^{\prime }}_{1} & {R^{\prime }}_{2} & \ldots & {R^{\prime }}_{n}\\ {G^{\prime }}_{1} & {G^{\prime }}_{2} & \ldots & {G^{\prime }}_{n}\\ {B^{\prime }}_{1} & {B^{\prime }}_{2} & \ldots & {B^{\prime }}_{n} \end{array}\right]$. According to the principle of least squares, the color temperature deviation calibration matrix (CCM) can be expressed as follows:

(5) $$CCM={\left(RGBM^{T}RGBM\right)}^{-1}RGBM^{T}RGBP$$

According to Equations (1)–(5), the color temperature deviation-calibrated stellar spectral type corresponding to the three fundamental colors $R_{i}(s)$, $G_{i}(s)$, and $B_{i}(s)$ is as follows:

(6) Ri(s)Gi(s)Bi(s)=CCM⋅a11a12a13a21a22a23a31a32a33⋅∫380780C1λ5(eC2λf(s)−1)⋅x¯(λ)dλ∫380780C1λ5(eC2λf(s)−1)⋅y¯(λ)dλ∫380780C1λ5(eC2λf(s)−1)⋅z¯(λ)dλ⋅1∫380780C1λ5(eC2λf(s)−1)⋅(x¯(λ)+y¯(λ)+z¯(λ))dλ

where $C_{1}$ is the first radiation constant and equals $3.741771\times 10^{-16}W\cdot m^{2}$; $C_{2}$ is the second radiation constant and equals $1.4387768\times 10^{-2}m\cdot K$.

### 3.3. Algorithm for Generating Stellar Maps with Color Temperature Information

Based on the stellar spectral type to RGB three-color conversion law and the color temperature deviation calibration, the stellar map presents the attitude information of the celestial coordinate system [39] (azimuth φ, pitch θ, roll λ) to obtain the stellar map pointing unit vector ${\left[x,y,z\right]}^{T}$ as follows:

(7) $$\left[\begin{array}{c} x\\ y\\ z \end{array}\right]=\left[\begin{array}{c} \cos\theta \cos\varphi \\ \cos\theta \sin\varphi \\ \sin\theta \end{array}\right]$$

The 3D coordinates of the star point [40] were screened according to the stellar map field of view (FOV), and the maximum coordinate values of the stellar map display device were set as $x_{\max}$ and $y_{\max}$. Then, the coordinates of the simulated star on the stellar map display device (x′, y′) were obtained as follows:

(8) $$\left\{\begin{array}{c} x^{\prime }=-\frac{y\cdot x_{\max}}{2x\tan(FOV/2)}\\ y^{\prime }=-\frac{z\cdot y_{\max}}{2x\tan(FOV/2)} \end{array}\right.$$

Subsequently, according to the stellar spectral type in the stellar catalog [41], Equations (5) and (6) were used to obtain the RGB triple colors of the OLED device corresponding to each star, thus completing the generation of the stellar map with color temperature information.

## 4. Experimentation and Discussion

### 4.1. Experimental Platform and Design

**Figure 3:**
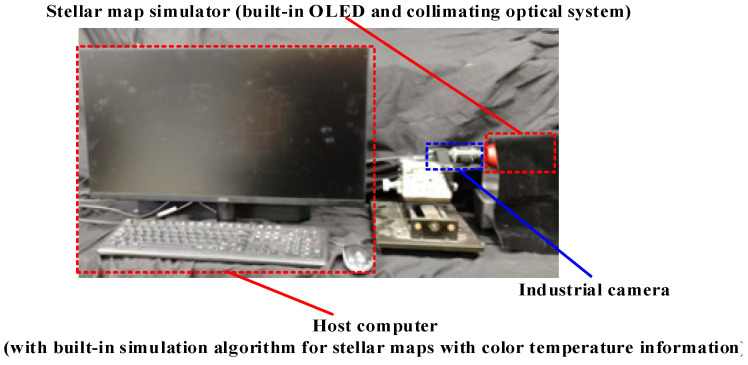
Experimental platform for semi-physical simulation of stellar maps with color temperature information.

In this study, the stellar map simulation algorithm with color temperature information was implemented on the host computer using the Visual Studio 2022 development tool based on the Microsoft Foundation Class framework. OpenCV image processing and Eigen matrix manipulation libraries were introduced during the development process. The stellar map simulation device was constructed using the Sony MicroOLED ECX331DB-6 with a resolution of 1024 × 768 and an 8-bit color depth OLED device, along with a collimated optical system with an FOV of 20° × 20° and a focal length of 35 mm. The MV-UB1000C by MindVision, which has 10 million pixels, was used as an industrial camera. The semi-physical simulation platform for stellar maps with color temperature information is shown in Figure 3.

Based on the stellar map simulation algorithm with color temperature information, two experimental phases were involved in this study: the pre-experiment and the validation experiment. The pre-experiment serves as the basis for the verification experiment. The specific experimental arrangement is summarized in Table 2.

### 4.2. Experimental Results of the Pre-Experimental Phase

The experiment on stellar map generation with color temperature information was divided into two parts: stellar spectral type to RGB triple-color conversion, and color temperature deviation calibration.

#### 4.2.1. Stellar Spectral Type to RGB Three-Color Conversion

**Figure 4:**
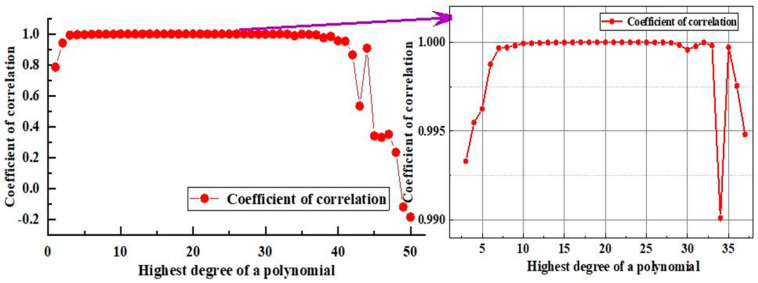
Number of polynomials vs. fitted correlation coefficient.

Polynomial fits from 1 to 50 times were conducted to obtain the optimal polynomial fit of the spectral type of the star to blackbody color temperatures. The fit correlation coefficient [36] was used to evaluate the polynomial fit. The relationship between polynomial counts and fit correlation coefficients is shown in Figure 4.

**Figure 5:**
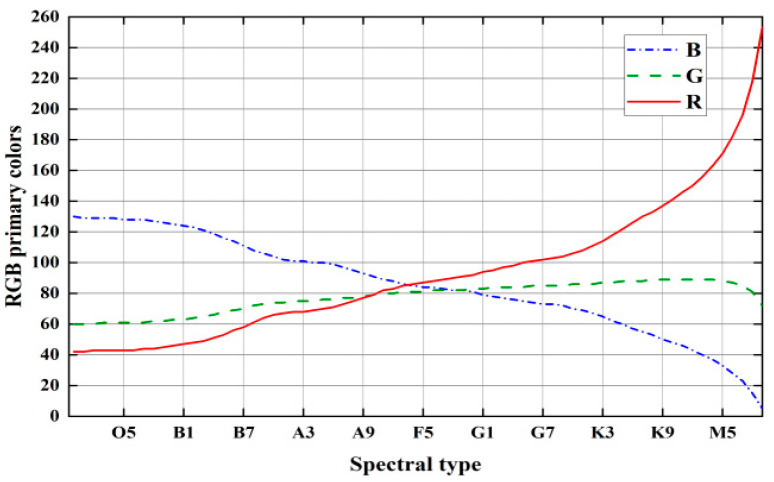
Stellar spectral type and RGB three-color scale relationship.

As shown in Figure 4, the fitting correlation coefficients were >0.99 for fitting times in the interval from 3 to 37, with the 22nd polynomial achieving the best fit. sRGB is the most widely used color space [37], and its color gamut is sufficient to cover the range of stellar colors [38]. Therefore, the sRGB color space was selected. The stellar spectral type and RGB triple-base color scale relations were obtained (Figure 5) according to Equations (2)–(4).

#### 4.2.2. Color Temperature Deviation Calibration

The 24-color standard color card was displayed on the OLED device (RGB three-color as $R_{i}$, $G_{i}$, and $B_{i}$). The industrial camera was used to obtain the image of the standard color card through the collimated optical system (RGB three-color $R_{i}^{\prime }$, $G_{i}^{\prime }$, and $B_{i}^{\prime }$), and according to Equation (5), the CCM was obtained as $\left[\begin{array}{rrr} 1.060828 & 0.008814 & -0.00608\\ -0.02595 & 0.992383 & -0.00513\\ -0.00894 & -0.00811 & 1.018448 \end{array}\right]$. Then, the RGB three-color of the standard color card was set after calibrating the color temperature deviation to $RC_{i}^{\prime }$, $GC_{i}^{\prime }$, and $BC_{i}^{\prime }$. The color difference of the 24 standard colors before and after the calibration of $\Delta E_{i}$ and $\Delta EC_{i}$ was determined as follows:

(9) $$\left\{\begin{array}{l} \Delta E_{i}=\sqrt{{\left(R_{i}^{\prime }-R_{i}\right)}^{2}+{\left(G_{i}^{\prime }-G_{i}\right)}^{2}+{\left(B_{i}^{\prime }-B_{i}\right)}^{2}}\\ \Delta EC_{i}=\sqrt{{\left(RC_{i}^{\prime }-R_{i}\right)}^{2}+{\left(GC_{i}^{\prime }-G_{i}\right)}^{2}+{\left(BC_{i}^{\prime }-B_{i}\right)}^{2}}\\ S_{\Delta E}=\sqrt{\frac{\sum_{i=1}^{24}{\left(\Delta E_{i}-\bar{\Delta E}\right)}^{2}}{23}}\\ S_{\Delta EC}=\sqrt{\frac{\sum_{i=1}^{24}{\left(\Delta EC_{i}-\bar{\Delta EC}\right)}^{2}}{23}} \end{array}\right.$$

**Figure 6:**
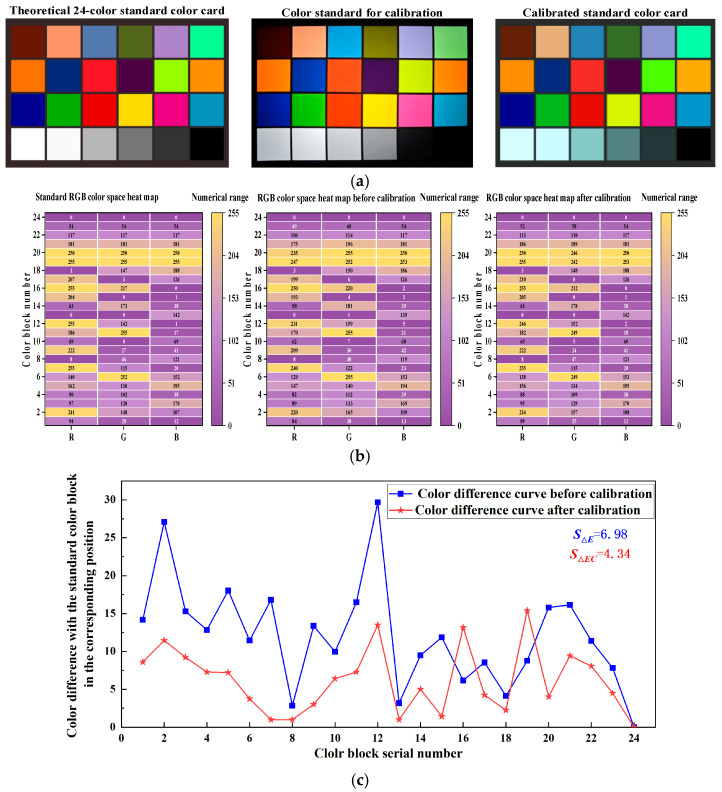
Color temperature deviation calibration results. (**a**) Stellar maps after color temperature deviation calibration. (**b**) Image and corresponding RGB three-color heat map. (**c**) Color difference between the 24 standard colors before and after calibration.

Here, $\Delta E_{i}$ is the sum of squares of the three primary color deviations of the standard color card before calibration, $\bar{\Delta E}$ is the average value of $\Delta E_{i}$, $\Delta EC_{i}$ is the sum of squares of the three primary color deviations of the standard color card after calibration, and $\bar{\Delta EC}$ is the average value of $\Delta EC_{i}$. Using the standard deviation of $\Delta E_{i}$ and $\Delta EC_{i}$, $S_{\Delta E}$ and $S_{\Delta EC}$, which indicates the overall simulation accuracy of the OLED device on the 24 standard colors of the color blocks before and after calibration, was calculated as 6.98 and 4.34, respectively. The theoretical 24-color standard color card, as well as the standard color card image and the corresponding RGB three-color heat map acquired by the industrial camera before and after calibration, are shown in Figure 6a,b, respectively. The color difference between the 24 standard color blocks before and after calibration is shown in Figure 6c. The $S_{\Delta EC}$ was smaller than $S_{\Delta E}$, implying that the color emitted by the OLED after calibration was closer to the standard color card.

### 4.3. Experimental Results of the Validation Experiment Phase

The simulation accuracy of stellar maps with color temperature information before and after calibrating the color temperature deviation was evaluated based on the mapping relationship between the stellar spectral type and RGB tri-color. This was achieved based on the following steps: (1) a mapping table of stellar spectral type ($SP_{i}$) and RGB tri-color ($R_{i},G_{i},B_{i}$) was established (notated as $Tab_{SP-RGB}$), and (2) an algorithm for identifying the spectral type of a stellar star point based on the look-up-table method was designed. In the algorithm, the principle of least squares was used to determine the spectral-type position (pos) matching the simulated stellar RGB three-color values (RS, GS and BS). Then, the corresponding spectral type of the star ($Stellar_{SP}$) was obtained. The discriminative formula of the stellar star-point spectral type identification algorithm is given as follows:

(10) $$\left\{\begin{array}{l} Tab_{SP-RGB}=\{SP_{i},R_{i},G_{i},B_{i}\}\;\;\;\;\;\;\;\;\;\;\;\;\;\;\;\;\;\;\;\;\;\;\;\;\;\;\;i\in \left[0,69\right]\\ pos=\{i\left|\min({\left(RS-R_{i}\right)}^{2}+{\left(GS-G_{i}\right)}^{2}+{\left(BS-B_{i}\right)}^{2})\right.\}\\ Stellar_{SP}=SP_{pos} \end{array}\right.$$

#### 4.3.1. Stellar Map Image Generation with Color Temperature Information

**Figure 7:**
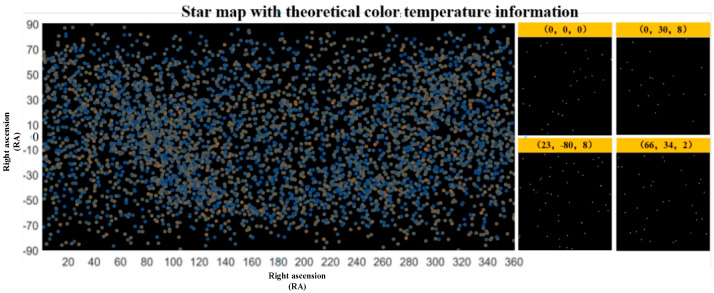
Theory of stellar maps with color temperature information.

Theoretical stellar maps with color temperature information were selected from the commonly used SAO catalog (5091 stars), with magnitudes ranging from 0 Mv to +6.0 Mv and above [39]. Three additional stellar maps with the attitudes (0, 30, 8), (23, −80, 8), and (66, 34, 2) were randomly generated using the stellar map simulation algorithm with color temperature information to obtain the central attitude stellar map (0, 0, 0) and prevent inconsistent experimental results. The overall distribution of the maps and the four attitudes of the stellar maps are shown in Figure 7.

#### 4.3.2. Semi-Physical Simulation of Stellar Maps with Color Temperature Information

**Figure 8:**
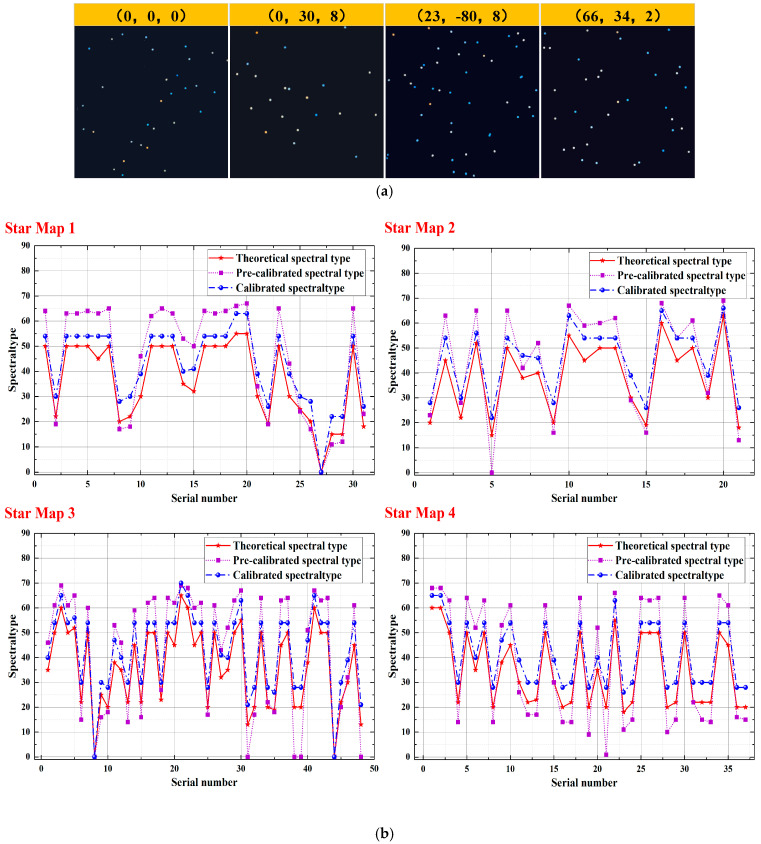
Simulation effect of the semi-physical simulation method of stellar maps with color temperature information. (**a**) Stellar maps after color temperature deviation calibration; (**b**) stellar maps after color temperature deviation calibration.

Four theoretical stellar maps with color temperature information were loaded onto the OLED device. The stellar maps with uncalibrated and calibrated color temperature deviations were successively captured using the industrial camera, and the spectral type simulation effect of the four maps was obtained using the stellar star-point spectral-type recognition algorithm. The color temperature deviation-calibrated stellar maps are shown in Figure 8a, and the spectral type simulation accuracy is shown in Figure 8b.

The maximum, average, and standard deviations of the spectral type simulations of the four stellar maps before and after calibration are summarized in Table 3. The maximum deviation of the simulated spectral types of the four stellar maps after calibration was 9, which is smaller than the metric scale of ten minor spectral types within a major stellar spectral type. This deviation was reduced by a factor of 1.89–2.11, demonstrating significant improvement in the accuracy of the stellar map spectral simulation. In addition, the average and standard deviations of the simulated spectral types of the four stellar maps after calibration were reduced by a factor of 1.29–1.73 and from 1.58 to 2.53, respectively. This indicates a substantial improvement in the accuracy of the spectral simulation of the stellar maps, as well as in the dispersion in the simulation of the overall color temperature information of the stellar maps.

### 4.4. Discussion and Comparison

The comparison between related studies on stellar map simulation and the present study is summarized in Table 4. Linghao Wu et al. [8] successfully simulated fainter stars; Schulz et al. [9] and Bin Zhao et al. [15] simulated the superposition of stellar maps and backgrounds; and Teague and Chahl [10] simulated stellar maps of motion-blurred stars. However, none of these studies could simulate color temperature. The simulators proposed in [13,14] can simulate color temperature in a wide range; however, only a single stellar color temperature can be simulated. The simulators developed in [18] and in this study can simulate the color temperature information of stars independently within the stellar map. However, in the simulator proposed in [18], only the energy ratios of four spectral intervals (ultraviolet, visible, mid-wave infrared, and long-wave infrared) can be adjusted, which provides a limited simulation of color temperature information. Conversely, the simulator developed in this study can cover the entire range of O–M types (the seven main spectral types) and the subtypes from 0–9 under each spectral type (a total of ten subtypes), totaling 70 stellar spectral types.

In summary, to the best of the authors’ knowledge, the proposed semi-physical simulation of stellar maps with color temperature information achieves independent controllability of the color temperature information of each star within the stellar map. Concurrently, the simulation range of stellar spectral types covers all 70 stellar spectral types of the HSC. However, this study presents a limitation in that the bit depth of the device is only 8 bits. Using a device with a higher bit depth would improve the correction ability of the proposed method. In future research, 16-bit color OLED display devices could be used to improve the degree of correction subdivision of the device and obtain better calibration results.

## 5. Discussion and Conclusions

This study aimed to overcome the lack of independent simulation methods for stellar color temperature in stellar maps and the complex structure of existing stellar map simulation systems with color temperature information. Therefore, a simplified stellar map simulation architecture based on OLED devices and a stellar map simulation algorithm with color temperature information were developed in this study. An experimental platform was developed, and two pre-experiments—stellar spectral type to RGB three-base color conversion and color temperature deviation calibration—were performed. Subsequently, four pairs of stellar maps were used to verify the semi-physical simulation effect of stellar maps with color temperature information. According to the experimental results, the findings are as follows:

1. The polynomial number of fits of the stellar spectral type to blackbody color temperature ranged from 3 to 37 correlation coefficients above 0.99, with the 22nd polynomial fit being the best. The standard deviation of the simulated color difference for the 24 standard colors was reduced by a factor of 1.6 after calibration using a 24-color standard color card.
2. The maximum deviation of the stellar spectral type simulations of the four stellar maps after color temperature bias calibration was better than 9, and the average deviation was better than 6.55, which are both smaller than the metric scale of 10 minor spectral types within a major stellar spectral type.
3. After the color temperature deviation calibration, the maximum deviation of the spectral type simulation of the four stellar maps was reduced by a factor of 1.89 to 2.11; the average deviation, by a factor of 1.29 to 1.73; and the standard deviation of the simulation deviation, by a factor of 1.58 to 2.53. These results indicate significant improvements in the limiting accuracy of the stellar map, the overall correctness of the simulation of color temperature information, as well as the dispersion.

In addition, compared with international studies, the developed simulator achieved independent control of the color temperature information of each star in the stellar map, and the spectral type simulation covered all 70 stellar spectral types of the HSC. The study findings provide a simulation method for the attitude determination algorithm using multi-dimensional information carried by the star sensor and establish a research basis for advancing the fields of remote sensing technology and spectral navigation.

In the future, devices [42] with better performance can be further investigated to improve the accuracy of stellar spectral-type simulation. The cosmic radiation background can be increased and the playback speed of the stellar map can be improved to help bring simulations closer to the actual stellar map observed in outer space. Color stellar map simulation technology provides strong technical support for the accuracy, reliability, and adaptability of remote sensing technology by optimizing the performance of remote sensing equipment, supporting the calibration of radiation characteristics of remote sensing data, and facilitating target recognition in complex environments. The innovative application of this technology not only promotes the development of the remote sensing field but also shows broad application prospects.

## Figures and Tables

**Table 1:** Rules for coding stellar spectral types.

Main Spectral Types	Encodings	Secondary Spectral Type	Encodings
TypO	0	Typ0	0
TypB	1	Typ1	1
TypA	2	Typ2	2
TypF	3	Typ3	3
TypG	4	Typ4	4
TypK	5	Typ5	5
TypM	6	Typ6	6
-	-	Typ7	7
-	-	Typ8	8
-	-	Typ9	9

**Table 2:** Experimental phases, names, and objectives.

Point	Name	Goal
Pre-experiment	Stellar spectral type to RGB triple-color conversion	The relationship between stellar spectral type and RGB triple-base color conversion is obtained
	Color temperature deviation calibration	The combined color temperature deviations of the OLED devices and collimated optical systems are calibrated
Verification experiment	Stellar map image generation with color temperature information	Theoretical stellar maps with color temperature information are generated to serve as a theoretical benchmark set to provide a baseline and reference for subsequent experiments
	Semi-physical simulation of stellar maps with color temperature information	Simulated stellar maps before and after calibrating the color temperature deviation are set as the uncalibrated control and experimental groups after calibration, respectively, to obtain the simulation effect of the semi-physical simulation method of stellar maps with color temperature information

**Table 3:** Maximum deviation, average deviation, and standard deviation for spectral-type simulations.

Star Atlas Serial Number	Maximum Deviation			Average Deviation			Standard Deviation		
	Before Calibration	After Calibration	Reduction Ration	Before Calibration	After Calibration	Reduction Ration	Before Calibration	After Calibration	Reduction Ration
Stellar Map 1	18	9	2	10.19	5.94	1.72	5.83	2.31	2.53
Stellar Map 2	18	9	2	5.9	4.58	1.29	5.70	3.61	1.58
Stellar Map 3	17	9	1.89	10.23	5.90	1.73	4.25	2.25	1.89
Stellar Map 4	19	9	2.11	10.19	6.55	1.56	4.75	1.96	2.42

**Table 4:** Comparison of related studies.

Author	Analog Stellar Map Features	Color Temperature Information Simulation Capability
Linghao Wu [8]	Stellar maps (fainter stars can be simulated)	-
Schulz et al. [9]	Stellar map (with controlled Gaussian background noise)	-
Teague and Chahl [10]	Stellar map (with motion blur simulation capability)	-
Qiang Liu [13]	Single star	3000–11,000 K color temperature simulation
Zhikun Yun [14]	Single star	2000–12,000 K color temperature simulation
Bin Zhao et al. [15]	Stellar map (with information on cosmic background radiation)	-
Songzhou Yang et al. [18]	Stellar map	The energy ratios of the four spectral intervals of each star are independently adjustable, without the ability to adjust the spectral type of the star
This study	Stellar map	The color temperature information for each star is independently adjustable, and the simulation covers all 70 stellar spectral types of the HSC

## Data Availability

The data underlying the results presented in this paper are not publicly available at this time but can be obtained from the authors upon reasonable request.

## References

[1] Zhang W., Yang Y., You W. (2022). Autonomous navigation method and technology implementation of high-precision solar spectral velocity measurement. Sci. China Phys. Mech. Astron..

[2] Wang Z., Liu S., Sun H. (2021). The influence of color temperature mismatch in star simulator on positioning accuracy and magnitude measurement by star sensor. J. Phys. Conf. Ser..

[3] Chaudhary A., Thapa B., Sodari T. (2022). Distribution of Dust Color Temperature, Planck’s Function, and Dust Mass around PSR J1240-4124. J. Nepal Phys. Soc..

[4] Huang S., Kang Z., Liu J. (2021). Accuracy analysis of spectral velocimetry for the solar Doppler difference navigation. IEEE Access.

[5] Zhou W., Liu Z., Sun Y. (2024). Bidirectional Littrow double grating interferometry for quadruple optical interpolation. Opt. Laser Technol..

[6] Niu Y., Wei X., Li J. (2022). Fast and robust star identification using color ratio information. IEEE Sens. J..

[7] Zhang W. (2020). Astronomical spectral velocimetry navigation technology and application considerations. Navig. Control.

[8] Wu L., Zhang G., Sun G. (2020). Optical engine optimization for faint starlight simulation systems. Opt. Commun..

[9] Schulz V.H., Marcelino G.M., Seman L.O. (2021). Universal verification platform and star simulator for fast star tracker design. Sensors.

[10] Teague S., Chahl J. (2022). Imagery synthesis for drone celestial navigation simulation. Drones.

[11] Trivellin N., Barbisan D., Ferretti M. (2016). Adaptive multi-wavelength LED star simulator for space life studies. Proc. SPIE.

[12] Jing J.L., Guoyu G.Y.Z., Jian J.Z. (2022). Spectral simulation method for calibration light source of transmissometers. Acta Opt. Sin..

[13] Liu Q., Zhang G., Zhang Y. (2023). Multi-color temperature and magnitude simulation for astronomical spectral velocity measurement. IEEE Access.

[14] Yun Z., Zhang Y., Liu Q. (2024). Research on the simulation method of a BP neural network PID control for stellar spectrum. Opt. Express.

[15] Zhao B., Zhang Y., Yang D. (2025). Playback method for dynamic star map simulation by fusing cosmic background radiation information. Measurement.

[16] Sun Q., Niu Z., Li Y., Wang Z. (2024). A Robust High-Accuracy Star Map Matching Algorithm for Dense Star Scenes. Remote Sens..

[17] Wang H., Yan Z., Mao X., Wang B., Liu X., Kang W. (2021). A new high-precision star map simulation model and experimental verification. J. Mod. Opt..

[18] Yang S., Zhang Y., Zhao B. (2024). Simulation method for multi-source information fusion space target. Opt. Express.

[19] Bai Y., Li J., Zha R. (2020). Catadioptric optical system design of 15-magnitude star sensor with large entrance pupil diameter. Sensors.

[20] Hong T., Li J., Chu D. (2023). Modulation approach of arbitrary linear polarization states of optical fields using single-beam coding for next-generation optical storage in glass. Opt. Laser Technol..

[21] Du Z., Sun G., Yang S. (2024). Research on ultraviolet-visible composite optical target simulation technology. Opt. Express.

[22] Ma M., Niu Y., Gao D., Li F., Shi G. (2025). Efficient Hyperspectral Video Reconstruction via Dual-Channel DMD Encoding. Remote Sens..

[23] Liu S., Zhang J., Zhang Y. (2023). Overall study of solar simulation optical system with large irradiated surface using free-form concentrator to improve uniformity. iScience.

[24] Wu L., Wang J., Sheng L. (2024). Research on polarization effect suppression method of weak starlight simulation device. Opt. Express.

[25] Gao X., Gan X., Chen Y. (2016). Study of digital high-precision multi-star simulator for multi-magnitude output. Int. J. Control Autom..

[26] Xu Q., Zhao C., Li X. Stellar radiation modeling and image simulation for airborne daytime star sensor. Proceedings of the IEEE International Conference on Signal and Image Processing (ICSIP).

[27] Saurova K., Shamro A., Alipbayev K. (2024). Research of methods to improve the accuracy of the star sensor for global navigation satellite system technology. Eng. Sci..

[28] Bell R.A., Gustafsson B. (1989). The effective temperatures and colours of G and K stars. Mon. Not. R. Astron. Soc..

[29] Catalbas M.C., Bernard Kobav M. (2022). Measurement of correlated color temperature from RGB images by deep regression model. Measurement.

[30] Han D., Lee M., Jang W. (2022). Two-color strip-patterned white OLEDs: Tunable color-temperature via pattern dimension control. Adv. Opt. Mater..

[31] Tian H.J., Chen T., Hu Y. (2021). Change of circadian effect with colour temperature and eye spectral transmittance at different ages. Lighting Res. Technol..

[32] Chaity M.D., van Aardt J. (2024). Exploring the Limits of Species Identification via a Convolutional Neural Network in a Complex Forest Scene through Simulated Imaging Spectroscopy. Remote Sens..

[33] Harre J.V., Heller R. (2021). Digital color codes of stars. Astron. Nachr..

[34] Bateni A., Susnar S.S., Amirfazli A. (2003). A high-accuracy polynomial fitting approach to determine contact angles. Colloids Surf. A Physicochem. Eng. Asp..

[35] Vicente E.G., Matesanz B.M., Rodríguez-Rosa M. (2023). Effect of correlated color temperature and S/P-ratio of LED light sources on reaction time in off-axis vision and mesopic lighting levels. LEUKOS.

[36] Burrow J.A., Jakachira R., Lemaster G. (2024). Smartphone tristimulus colorimetry for skin-tone analysis at common pulse oximetry anatomical sites. arXiv.

[37] Kahu S.Y., Raut R.B., Bhurchandi K.M. (2019). Review and evaluation of color spaces for image/video compression. Color Res. Appl..

[38] Galteri L., Ferrari C., Kollias S. (2022). Color and texture analysis of textiles using image acquisition and spectral analysis in calibrated sphere imaging system-I. Electronics.

[39] Bian H., Li A., Ma H., Wang R. (2024). Navigation coordinate system. Essentials of Navigation: A Guide for Marine Navigation.

[40] Yuan H., Li D., Wang J. (2022). A Robust Star Identification Algorithm Based on a Masked Distance Map. Remote Sens..

[41] Yi H., Yan G. (2010). Preliminary research on color simulation of stars in a star simulator. Opto-Elec Eng..

[42] Zhang J., Yu S., Wang Y. (2024). Research on manufacturing technology of nanoimprinted grating. J. Manuf. Process..

